# Preclinical evaluation of PSMA expression in response to androgen receptor blockade for theranostics in prostate cancer

**DOI:** 10.1186/s13550-018-0451-z

**Published:** 2018-10-29

**Authors:** Katharina Lückerath, Liu Wei, Wolfgang P. Fendler, Susan Evans-Axelsson, Andreea D. Stuparu, Roger Slavik, Christine E. Mona, Jeremie Calais, Matthew Rettig, Robert E. Reiter, Ken Herrmann, Caius G. Radu, Johannes Czernin, Matthias Eiber

**Affiliations:** 1 Department of Molecular and Medical Pharmacology, David Geffen School of Medicine at UCLA, Los Angeles, CA USA; 2 Department of Nuclear Medicine, Universitätsklinikum Essen, Essen, Germany; 3 Lund University, Lund, Sweden; 4 Department of Urology, David Geffen School of Medicine at UCLA, Los Angeles, CA USA; 5 Department of Nuclear Medicine, Klinikum rechts der Isar, Technical University of Munich, Munich, Germany; 6 University of California at Los Angeles, Ahmanson Translational Imaging Division, 10833 Le Conte Ave, 200 Medical Plaza, Ste. B114-61, Los Angeles, CA 90095-7370 USA

**Keywords:** PSMA, Prostate cancer, ^68^Ga-PSMA PET/CT, Androgen receptor blockade, Radioligand therapy

## Abstract

**Background:**

Prostate-specific membrane antigen (PSMA)-directed radioligand therapy (RLT) is a promising yet not curative approach in castration-resistant (CR) prostate cancer (PC). Rational combination therapies may improve treatment efficacy. Here, we explored the effect of androgen receptor blockade (ARB) on PSMA expression visualized by PET and its potential additive effect when combined with ^177^Lu-PSMA RLT in a mouse model of prostate cancer.

**Methods:**

Mice bearing human CRPC (C4-2 cells) xenografts were treated with 10 mg/kg enzalutamide (ENZ), with 50 mg/kg bicalutamide (BIC), or vehicle (control) for 21 days. PSMA expression was evaluated by ^68^Ga-PSMA11 PET/CT and quantified by flow cytometry of tumor fine needle aspirations before treatment and on days 23, 29, 34, and 39 post-therapy induction. For the RLT combination approach, mice bearing C4-2 tumors were treated with 10 mg/kg ENZ or vehicle for 21 days before receiving either 15 MBq (84 GBq/μmol) ^177^Lu-PSMA617 or vehicle. DNA damage was assessed as phospho-γH2A.X foci in tumor biopsies. Reduction of tumor volume on CT and survival were used as study endpoints.

**Results:**

Tumor growth was delayed by ARB while ^68^Ga-PSMA11 uptake increased up to 2.3-fold over time when compared to controls. ABR-induced upregulation of PSMA expression was confirmed by flow cytometry. Phospho-γH2A.X levels increased 1.8- and 3.4-fold at 48 h in response to single treatment ENZ or RLT and ENZ+RLT, respectively. Despite significantly greater DNA damage and persistent increase of PSMA expression at the time of RLT, no additional tumor growth retardation was observed in the ENZ+RLT group (vs. RLT only, *p* = 0.372 at day 81). Median survival did not improve significantly when ENZ was combined with RLT.

**Conclusion:**

ARB-mediated increases in PSMA expression in PC xenografts were evident by ^68^Ga-PSMA11 PET imaging and flow cytometry. ^177^Lu-PSMA617 effectively decreased C4-2 tumor size. However, while pre-treatment with ARB increased DNA damage significantly, it did not result in synergistic effects when combined with RLT.

**Electronic supplementary material:**

The online version of this article (10.1186/s13550-018-0451-z) contains supplementary material, which is available to authorized users.

## Introduction

The therapeutic landscape of castration-resistant (CR) prostate cancer (PC) is changing. Recent introduction of second-line androgen receptor blockade (ARB) has improved the quality of life and patient survival. For instance, enzalutamide (ENZ) increased median survival by 2.2 months [1] and 4.8 months [2], respectively, when compared to placebo.

The development of highly selective radiolabeled ligands binding to prostate-specific membrane antigen (PSMA) opened up an additional therapeutic option (PSMA-targeted radioligand therapy (RLT)) [3] that has been exploited in more than 1000, mainly CRPC, patients worldwide, often in combination with basic ARB.

ARB and androgen withdrawal transcriptionally upregulate PSMA expression and enhanced the effect of a PSMA-antibody drug conjugate [4–8]. Higher PSMA levels might lead to increased binding of PSMA-targeting agents and thus delivery of higher RLT tumor doses. However, the longitudinal effects of ARB-mediated increases in PSMA on the therapeutic efficacy of PSMA-targeted radioligand therapy have not been investigated.

Accordingly, we designed this study to (i) explore the kinetics of ABR-induced changes in PSMA-expression and (ii) investigate the potential additive effects of ARB (ENZ) and ^177^Lu-PSMA617 RLT in a mouse model of PC.

## Methods

### Cell culture

C4-2 (kind gift of G. Thalmann, University of Bern, Switzerland) is a human prostate cancer cell line reflecting androgen-independent disease [9]. C4-2 expresses robust and high levels of PSMA at the cell surface [10]. Cells were cultured in Rosewell Park Memorial Institute (RPMI) 1640 medium supplemented with 10% fetal bovine serum at 37 °C and 5% CO_2_. Cells were routinely checked for mycoplasma contamination using the MycoAlert PLUS mycoplasma detection kit (Lonza).

### Mice

**Fig. 1:**
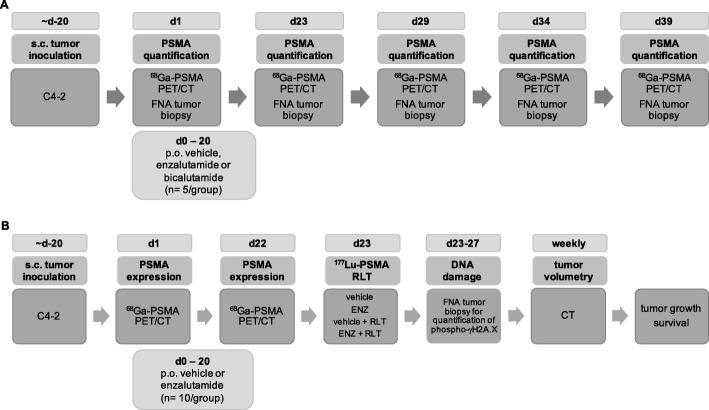
Experimental design. **a** Investigation of ARB (ENZ vs. bicalutamide (BIC) vs. vehicle)-mediated PSMA upregulation in C4-2 xenografts. **b** Testing efficacy of pre-treatment with ENZ before PSMA-RLT in C4-2 tumors. DNA damage was assessed 4 h, 2 and 4 days post RLT by flow cytometric quantification of phospho-γH2A.X in FNA tumor biopsies

All animal studies were approved by the UCLA Animal Research Committee (ARC; # 2005-090). Intact (non-castrate) male, 6-week-old Scid mice were housed under pathogen-free conditions. Water and food were provided ad libitum. Mice were injected subcutaneously with 5 × 10^6^ cells in matrigel into the shoulder region. Tumor growth was monitored by computed tomography (CT). Animals were sacrificed upon reaching any of the termination criteria specified in the ARC protocol, including apathy, ulceration, severe weight loss, or other signs of deteriorating condition. The study protocol is outlined in Fig. 1.

### Positron emission tomography/computed tomography (PET/CT)

^68^Ga-PSMA11 (5 μg precursor) was obtained from the UCLA Biomedical Cyclotron. Static PET/CT images were acquired 60 min after the tail vein injection of approximately 1.1 MBq ^68^Ga-PSMA11 using the pre-clinical Genisys 8 PET/CT scanner [11] (Sofie Biosciences). Image acquisition time was 10 min. Attenuation-corrected images were reconstructed using maximum-likelihood expectation maximization with 60 iterations. The following parameters were applied for CT imaging: 40 kVp, 190 mA, 720 projections, and 55-ms exposure time per projection.

Tumors were delineated on ≥ 7 axial CT images and were encompassed in a volume of interest. Tumor volume was calculated using OsiriX Imaging Software (Version 3.9.3; Pixmeo SARL). The volume of interest from CT was copied to the PET dataset to calculate the mean and maximum percentage injected activity/gram (%IA_mean_/g and %_IAmax_/g).

### Fine needle aspiration (FNA) biopsy of mouse tumors

For serial monitoring of PSMA expression, tumor samples were acquired by fine needle aspiration biopsy [10, 12, 13]. Syringes were filled with 500 μl PBS, and a 25-G needle was attached. To account for tumor heterogeneity, tumor material was aspirated at three different sites within the same tumor and flushed out as one sample. Cell suspensions were used for flow cytometric analysis of PSMA or phospho-γH2A.X expression.

### Flow cytometry

Single-cell suspensions were stained with an anti-hPSMA-APC antibody (clone REA408; 2.5 μl/test; Miltenyi Biotech) for 30 min at 4 °C. Samples were fixed and permeabilized using the Cytofix/Cytoperm kit from BD and stained with an anti-phospho-γH2A.X-FITC antibody (1:800, 20 min, RT, dark; Millipore) and DAPI to analyze DNA damage. Samples were measured on a 5-laser LSRII cytometer (Beckton Dickinson) and analyzed using FlowJo software (Tri Star).

### ARB

Mice were treated with 10 mg/kg ENZ [4], with 50 mg/kg BIC (both drugs: MedChem Express), or vehicle (1% labrasol/1% Tween-80/30% transcutol/68% PEG-200) per oral gavage for 21 days.

### Radioligand therapy with ^177^Lu-PSMA617

Non-carrier-added lutetium-177 trichloride was obtained from isotope technologies Garching GmbH, Germany. The precursor PSMA-617 was obtained from ABX GmbH, Germany. Radiolabeling was performed at the UCLA Biomedical Cyclotron Facility. Mice received a single injection of ^177^Lu-PSMA617 (15 MBq; 84 GBq/μmol) or 0.9% saline (vehicle control) per tail vein injection, respectively. The activity of ^177^Lu-PSMA617 was chosen based on a preliminary dose escalation study. In C4-2 tumors, 15 MBq ^177^Lu-PSMA617 was the lowest activity we tested resulting in a significant tumor growth inhibition compared to the control group. Therefore, 15 MBq ^177^Lu-PSMA617 were considered to be a sub-optimal dose that is suitable to investigate potential synergistic treatment effects in this model.

### Experimental design

#### Assessing temporal changes in PSMA expression

We investigated ARB-induced effects on PSMA cell surface expression (Fig. 1a). C4-2 tumors were established in Scid mice (5 mice/treatment group, see below). At day 19 ± 1 (baseline), PSMA expression was assessed by ^68^Ga-PSMA11 uptake by PET/CT and flow cytometry of FNA tumor biopsies. Mice were then randomized into three groups receiving enzalutamide, bicalutamide, and vehicle, respectively, for 21 days. ENZ and BIC are clinically used for treatment of mCRPC and castration sensitive PC, respectively. To investigate ARB-induced PSMA expression kinetics, changes in PSMA levels were assessed by ^68^Ga-PSMA11 PET/CT and flow cytometry on days 23, 29, 34, and 39 after tumor induction. These time points were chosen based on our preliminary data indicating that maximal PSMA induction could be observed ~ 25 days after start of ARB (i.e., ~ 3 days after cessation of ARB treatment; Additional file 1: Figure S1).

#### Investigating the efficacy of combined ARB and ^177^Lu-RLT

The results from the first part of the study indicated that a 21-day ENZ regimen increased C4-2 tumor PSMA expression at least up to days 34–39 peaking around days 23–29 (see result below). Based on these findings, we tested the potential additive effects of ENZ and ^177^Lu-PSMA617 RLT in C4-2 tumors as follows (Fig. 1b): C4-2 tumors were induced in Scid mice. On day 20, mice were randomized into two groups treated with ENZ (*n* = 20) or vehicle (*n* = 20) for 21 days (analogous to the above study). Two days after the end of treatment (day 23, i.e., at the time of maximal ENZ-induced PSMA levels) mice were further randomized into RLT or vehicle subgroups (*n* = 10 mice/subgroup). DNA damage was assessed 4 h, 48 h, and 96 h post RLT by flow cytometric quantification of phospho-γH2A.X in FNA tumor biopsies. Treatment efficacy was determined by tumor volume changes on serial CT and by survival.

All interventions were conducted in a blinded fashion.

### Statistics

Data were analyzed by readers blinded to the type of intervention. Data are presented as mean ± standard deviation (SD) or median and range as appropriate. Two-way ANOVA without correction for multiple comparisons (Fisher’s LSD) was used to compare different groups. Survival was analyzed by the Kaplan-Meyer method and one-way ANOVA without correction for multiple testing (Fisher’s LSD). *P* < 0.05 was considered statistically significant. GraphPad Prism (version 7, GraphPad Software, Inc.) was used for all statistical analyses.

## Results

### PSMA expression in C4-2 xenografts by ^68^Ga-PSMA11 PET imaging

**Fig. 2:**
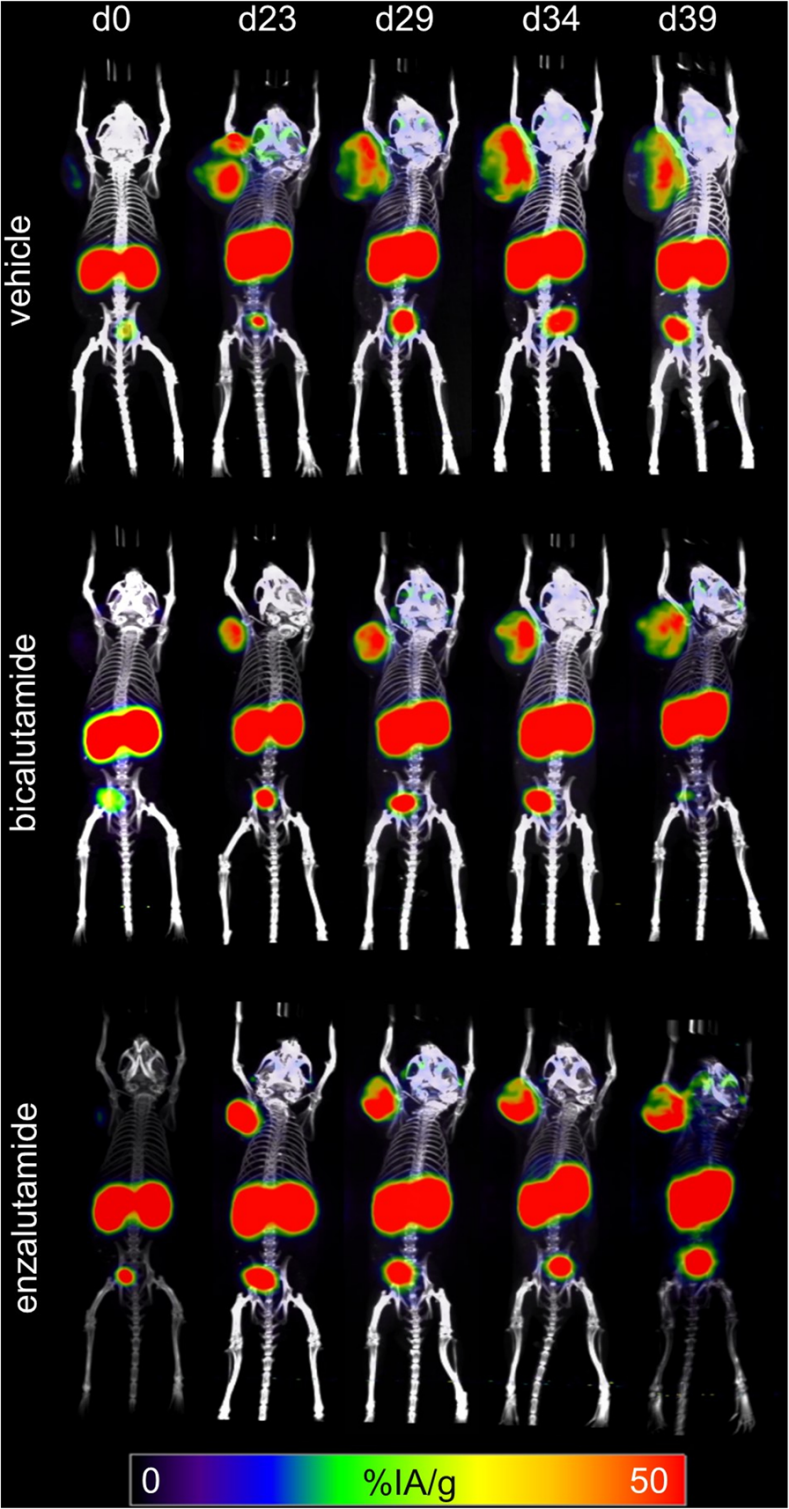
^68^Ga-PSMA11 PET/CT. C4-2 tumor-bearing mice were imaged with ^68^Ga-PSMA11 PET/CT on days 0, 23, 29, 34, and 39 post start of therapy with vehicle (top row), BIC (middle), or ENZ (bottom row). Representative PET/CT images of 1 mouse per treatment group are shown

**Fig. 3:**
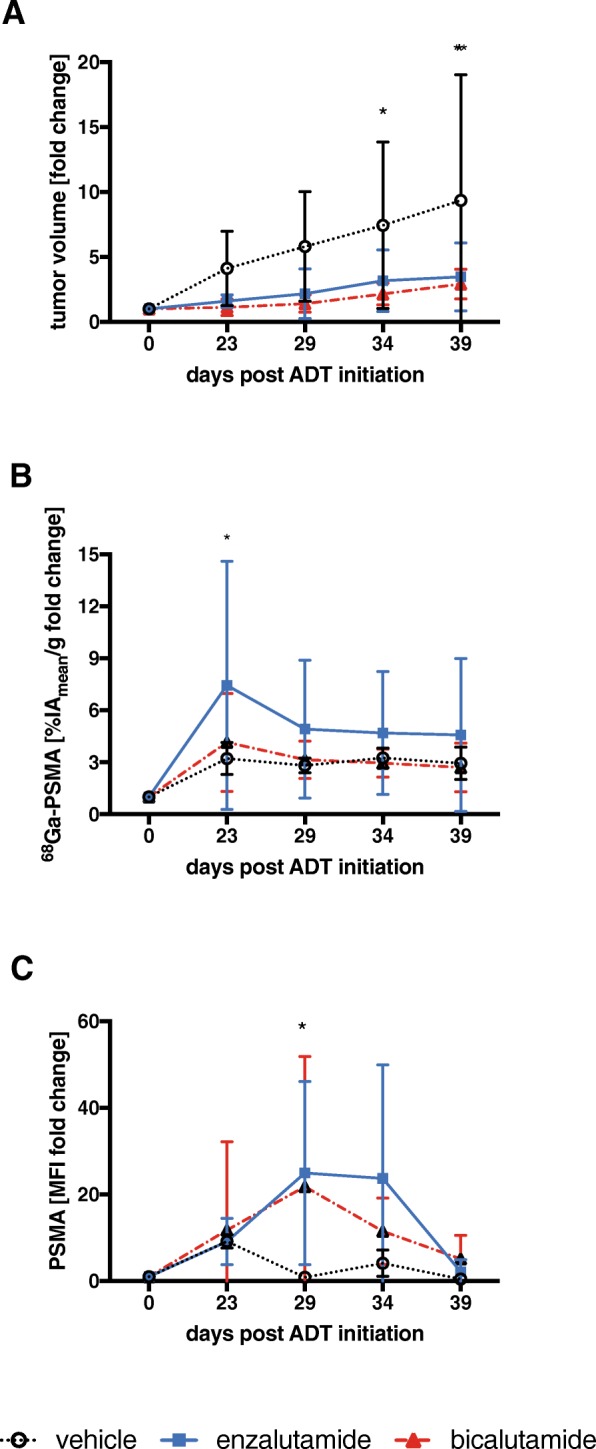
ARB increases PSMA at the tumor cell surface. Tumor volume of C4-2 (**a**) tumors was determined by CT (ENZ vs. vehicle, *p* = 0.05; BIC vs. vehicle, *p* = 0.02). ^68^Ga-PSMA11 PET signal (%IA_mean_/g) in C4-2 (**b**) tumors (ENZ vs. vehicle, *p* = 0.02; BIC vs. vehicle, *p* = n.s.). PSMA mean fluorescent intensity (MFI) is shown for each group (ENZ vs. vehicle, *p* = 0.03; BIC vs. vehicle, *p* = n.s.) (**c**). The fold-change during ARB treatment, related to the pre-treatment volume is shown. Data are shown as mean ± SD. Asterisks indicate significance

Tumor growth was delayed by ARB in the C4-2 model (at day 39, *p* = 0.009 (ENZ) and *p* = 0.005 (BIC); Fig. 3a, Additional file 2: Figure S2). ^68^Ga-PSMA11 tumor uptake tended to increase over time in all mice, peaking at day 23 and slightly decreasing thereafter yet remaining above baseline throughout the observation period (days 34–39 post ARB induction). The mean fold increase in %IA_mean_/g (compared to baseline) at day 23 was 3.2 ± 0.9 (vehicle), 7.4 ± 7.2 (ENZ; *p* = 0.018 compared to vehicle), and 4.2 ± 12.8 (BIC; *p* = 0.591), respectively (Fig. 3b; Additional file 3: Table S1). For temporal changes in PSMA expression as estimated from ^68^Ga-PSMA 11 images, see Fig. 2 and 3b.

### PSMA expression by flow cytometry

PSMA expression per cell was quantified using flow cytometry from tumor biopsies obtained after each PET/CT scan. Relative PSMA levels in C4-2 tumors peaked at day 29 (MFI compared to baseline) and were 0.9 ± 0.3 (vehicle), 25.0 ± 21.2 (ENZ; *p* = 0.032 compared to vehicle), and 21.8 ± 30.1 (BIC; *p* = 0.051 compared to vehicle) (Fig. 3c; Additional file 3: Table S1).

### Efficacy of RLT combined with ARB

As increased PSMA expression might lead to increased tumor RLT doses, we pre-treated mice bearing C4-2 tumors with ENZ before ^177^Lu-PSMA617 treatment. We selected ENZ over BIC due to its stronger PSMA induction.

Please note that RLT was given 2 days after the last ENZ dose (day 23), as our experiments showed that PSMA expression remains elevated for 14–19 days after ENZ treatment termination (Figs. 2 and 3; Additional file 1: Figure S1).

### Pre-treatment with ENZ enhanced RLT-induced DNA damage

**Fig. 4:**
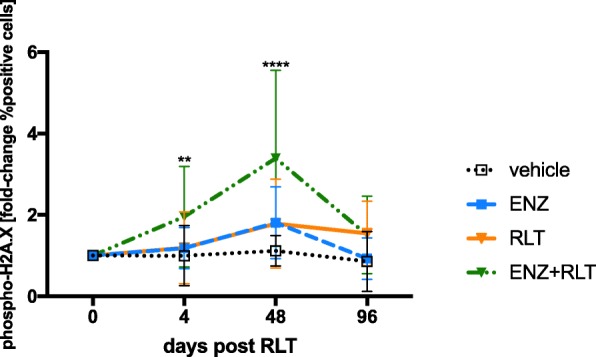
Pre-treatment with ENZ increases ^177^Lu-PSMA617-induced DNA damage. Phospho-γH2A.X levels were flow cytometrically quantified in FNA tumor biopsies 4 h, 48 h, and 96 h following administration of ^177^Lu-PSMA617 or vehicle, respectively. The fold-change in phospho-γH2A.X compared to baseline (day before RLT) is shown as mean ± SD. Asterisks indicate significance in ENZ+RLT-treated tumors compared to baseline (ENZ+RLT: 0 vs. 4 h, *p* = 0.003; 0 vs. 48 h, *p* < 0.0001)

Treatment with ENZ, RLT, or the combination of ENZ and RLT (ENZ+RLT) induced DNA damage in C4-2 tumors. The number of phospho-γH2A.X foci peaked 48 h post-administration of RLT (Fig. 4; Additional file 4: Table S2).

When compared to vehicle, phospho-γH2A.X levels increased 1.8-fold at 48 h in both the ENZ-only and RLT-only groups and 3.4-fold in ENZ+RLT group (ENZ-only: *p* = 0.017; RLT-only: *p* = 0.051; ENZ+RLT: *p* < 0.0001). Phospho-γH2A.X levels were highest in the ENZ+RLT group (*p* ≤ 0.0005 vs. ENZ-only and RLT-only). Phospho-γH2A.X foci decreased to baseline in the ENZ, RLT, and ENZ+RLT groups after 96 h.

### ENZ-induced PSMA upregulation and DNA damage did not translate into additional tumor growth retardation

**Fig. 5:**
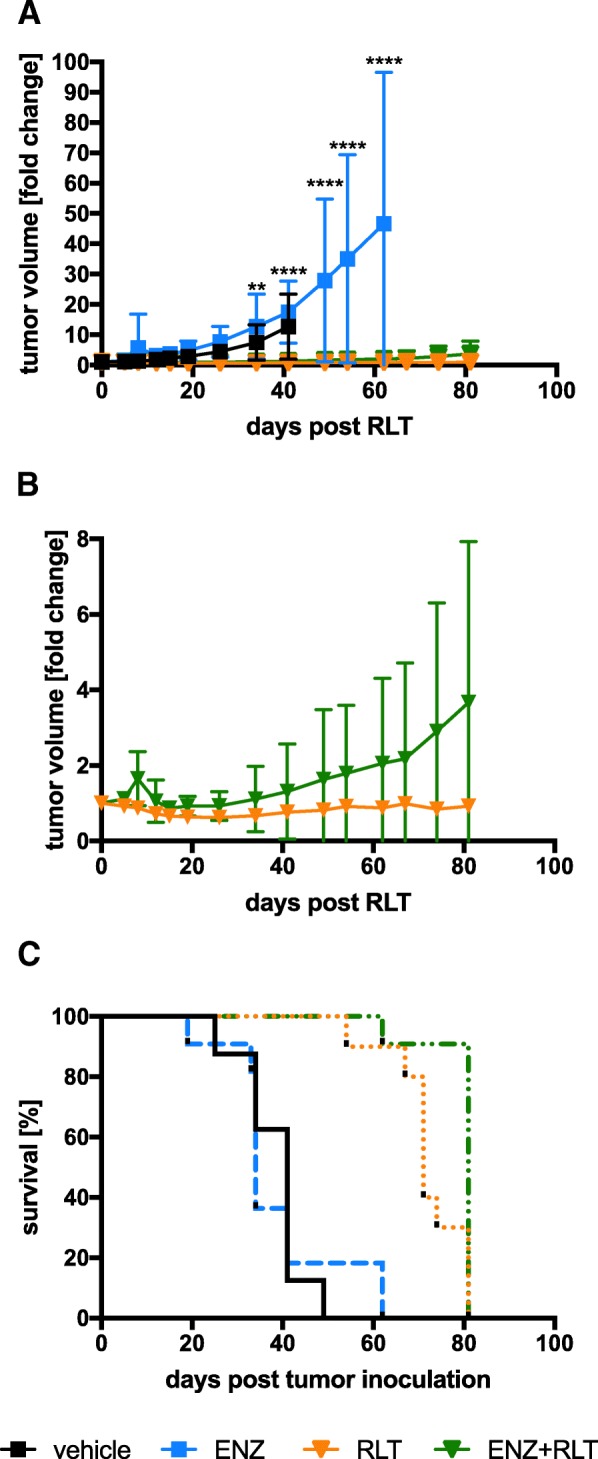
Efficacy of combined ARB and ^177^Lu-PSMA617 radioligand therapy. Tumor volume was determined by CT. The fold-change in tumor volume after RLT (day 1) administration related to the tumor volume immediately prior to RLT (day 0) is shown. The last dose of ENZ was given at day 0. Data are represented as mean ± SD. **a** Tumor volume change in all groups. Asterisks indicate significance in vehicle and ENZ groups vs. RLT and ENZ+RLT groups, respectively (****p < 0.0001; **p < 0.01). **b** Tumor volume change in the RLT and ENZ+RLT groups (*p* = not significant). **c** Kaplan-Meyer plot for survival

RLT retarded tumor growth when compared to the groups receiving vehicle or ENZ only (Fig. 5a; Additional file 2: Figure S2 and Additional file 5: Table S3). Tumor growth differed significantly starting on day 34 (vehicle vs. RLT: *p* = 0.003; vehicle vs. ENZ+RLT: *p* = 0.005; ENZ vs. RLT or ENZ+RLT: *p* < 0.0001; vehicle vs. ENZ: *p* = 0.027). Pre-treatment with ENZ before RLT did not retard tumor growth further within our observation period (RLT vs. ENZ+RLT, *p* = 0.372 at day 81; Fig. 5a, b).

### Conditioning with ENZ before RLT does not significantly improve survival

Median survival following RLT was 34 (range 19–62), 41 (range 25–49), 71 (range 54–81), and 81 (range 62–81) days in the ENZ-only, vehicle, RLT-only, and ENZ+RLT groups, respectively (Fig. 5c). Thus, RLT alone or combined with ENZ prolonged survival significantly when compared to vehicle (*p* < 0.0001). However, combining RLT with ENZ only tended to improve survival in the ENZ+RLT group when compared to RLT alone. The mean survival difference was 10 days (RLT vs. ENZ+RLT, *p* = 0.08). It should be noted that a reliable survival analysis could not be performed as on day 81 all remaining mice (3/10 in the RLT group; 8/11 in the ENZ+RLT group) had to be sacrificed per veterinarian instruction, due to worsening mouse condition (e.g., ulceration of tumor, tumor size).

## Discussion

The major hypothesis tested in our study was whether increased levels of PSMA as induced by ARB result in enhanced tumor targeting by ^177^Lu-PSMA617. As androgen receptor suppresses PSMA transcription [14], its inhibition with enzalutamide might lead to PSMA upregulation and, consequently, to a more effective treatment of tumors with ^177^Lu-PSMA617.

We therefore investigated in a mouse model of prostate cancer (i) the effect of ARB on PSMA expression over time and (ii) the effects of ARB on the efficacy of ^177^Lu-PSMA617 RLT. Our results indicate that a 21-day regimen of ENZ increases PSMA expression in C4-2 xenografts that peaks around days 23–29 and is maintained even after cessation of ENZ administration. However, ENZ-induced PSMA expression does not retard tumor growth more profoundly than ^177^Lu-PSMA617 alone. Furthermore, survival was not significantly improved.

First, we established ARB regimens with BIC or ENZ which effectively delayed tumor growth and induced a transient increase in PSMA expression in human prostate cancer xenografts in vivo. PSMA levels peaked 2–8 days (days 23–29 after ENZ start) after cessation of ARB treatment. Thus, when treated with ENZ for 21 days, PSMA levels remained above baseline for another 13–18 days (days 34–39 after ENZ start). This provided the rationale for administrating RLT on day 23 (see below).

The increases in ^68^Ga-PSMA11 tumor uptake in ARB-treated animals over controls were small (max. ~ 2-fold). Both the small increment and the PSMA kinetics corroborate and extend previous studies investigating the effect of androgen receptor inhibition on PSMA levels and PSMA-targeted imaging [4–7]. These studies reported enhanced PSMA expression in PC xenograft mouse models (i) upon androgen deprivation (6 days treatment) using ^64^Cu-J591 PET on day 7 (an antibody targeting the extracellular domain of PSMA) [4] and (ii) following orchiectomy or a 7-day regimen of apalutamide using ^68^Ga-PSMA11 PET on day 7 [5]. The latter study also reports increased PSMA levels in a patient following a 30-day ARB therapy. Only one study investigated PSMA levels after ARB (ENZ) removal in vitro [7]. Following a 21-day treatment with ENZ, PSMA levels peaked around day 21 and remained elevated (compared to untreated cells) 4–7 days after ENZ discontinuation. Importantly, this study demonstrated increased efficacy of PSMA antibody-drug conjugates with increasing PSMA expression.

Second, RLT and ENZ+RLT significantly delayed tumor growth and improved survival (compared to ENZ-only and vehicle). In addition, pre-treatment with ENZ led to more profound DNA damage (phospho-γH2A.X foci) compared to RLT alone. This is in line with in vitro studies reporting that treatment with ENZ combined with irradiation [15, 16] or the *p*oly (*A*DP-*R*ibose) *p*olymerase inhibitor olaparib [17] increased the number of phospho-γH2A.X foci resulting from either treatment (X-ray or olaparib) alone.

Although this would be expected to improve therapeutic efficacy, pre-treatment with ENZ did not result in additional tumor growth retardation when RLT was compared to ENZ+RLT. An accurate survival analysis was not possible as on day 81 all remaining mice (3/10 in the RLT group; 8/10 in ENZ+RLT) had to be sacrificed (compare last paragraph of result section).

While ENZ efficiently reduced tumor growth (Fig. 3a), tumors rapidly regrew in the ENZ-only group after termination of ENZ treatment (Fig. 5a shows tumor size change from the time after completion of ENZ and before RLT). This might suggest that ARB is only effective during treatment. However, as shown in Figs. 2 and 3b and c, increased PSMA expression persisted after cessation of ENZ up to day 34, i.e., almost 2 weeks after end of ENZ therapy. In fact, PSMA levels peaked between 23 and 29 days after start of enzalutamide treatment (Fig. 3b, c). ^177^Lu-PSMA617 radioligand therapy was performed on day 23 (i.e., 2 days after the last dose of ENZ), at a time when ENZ-induced PSMA expression peaked. ^177^Lu-PSMA617 binds to PSMA-expressing tumors within hours [18]. It is then rapidly internalized [19]. Unbound ^177^Lu-PSMA617 is excreted via the kidneys. Therefore, we ascertained high PSMA expression levels at the time of RLT administration. Maintaining high PSMA expression beyond the time of RLT is thus likely less (if at all) relevant for RLT efficacy. It remains unclear whether ARB-induced increases in PSMA expression might have been insufficient to enhance RLT efficacy. While we did not perform biodistribution studies to determine if the increased PSMA levels led to a higher tumor uptake of ^177^Lu-PSMA617, preclinical evaluation of ^68^Ga-PSMA11/^177^Lu-PSMA617 by Umbricht et al. demonstrated very similar uptake and internalization of the ^68^Ga- and ^177^Lu-ligand, respectively, by PC-3-PIP cells [20]. Likewise, the %IA/g in PC3-PIP tumors was comparable for ^68^Ga-PSMA11 and ^177^Lu-PSMA617. In a similar study, Weineisen et al. [21] found that uptake and internalization of ^68^Ga/^177^Lu-PSMA I&T—a similarly used theranostic pair of PSMA ligands—into LNCaP cells was almost identical. Lastly, SUV_mean_ on ^68^Ga-PSMA11 PET/CT images correlated with absorbed dose in men with mCRPC [22]. Therefore, we propose that the uptake of ^177^Lu-PSMA617 into C4-2 tumors can be inferred from the uptake of ^68^Ga-PSMA11. Our data (Figs. 2 and 3) thus suggest that tumors conditioned with ENZ took up more ^177^Lu-PSMA617 than tumors without ENZ-induced PSMA upregulation. However, the actual absorbed dose of ^177^Lu-PSMA617 in tumor or organs cannot be calculated based on ^68^Ga-PSMA11 PET images, because the PET images only give information about the tracer activity. Future studies will need to define the relationship between the degree of target expression, tumor-absorbed dose, and RLT efficacy.

An alternative or additional explanation for the lack of synergy is based on the notion that AR signaling has been implicated in DNA damage repair [23–25]. In the setting of RLT, AR-triggered repair might protect tumor cells from cell death; its impairment by ARB could thus synergize with RLT. However, radiation causes the activation of several DNA damage repair pathways that act in parallel [26]. As we did not observe an enhancement of RLT by ARB, either (i) impairment of AR-triggered repair might not be a critical determinant of RLT efficacy on its own or (ii) the ENZ-mediated AR blockade ceased with discontinuation of the ENZ administration. The latter might imply the need for conditioning plus continuation of ARB under RLT to first increase PSMA expression and, subsequently, block repair of DNA damage once it occurs.

Finally, it is possible that surgical castration or castration plus ENZ would have had a greater impact on AR signaling and, consequently, PSMA expression, tumor growth, and (AR-mediated) DNA damage repair. However, Evans et al. and Hope et al. directly compared the impact of surgical castration vs. treatment with anti-androgen (10 mg/kg ENZ and 10 mg/kg apalutamide, respectively) on PSMA levels and tumor growth [4, 5]. While both interventions decreased tumor growth to a similar extent, upregulation of PSMA was stronger following treatment with anti-androgens.

Combination therapies will be required to enhance the effectiveness of RLT. For instance, a current randomized phase III trial investigates the combination of abiraterone and the alpha emitter Radium 223 (NCT02043678) in mCRPC. Another prospective trial (e.g., ANZCTR12615000912583) combines second-line ARB in conjunction with ^177^Lu-PSMA617. Finally, a recently started trial in Australia combines RLT with DNA damage response inhibitors (NCT03307629).

While we failed to show improved tumor growth retardation with ENZ+RLT, a beneficial survival benefit cannot be ruled out with certainty. First, mice had to be sacrificed on day 81, mostly due to tumor ulceration. Thus, it is possible that survival benefits in the combination therapy group may have been missed. Secondly, responses to RLT combinations may differ between the current and other murine models. We selected the current model as C4-2 reflects the clinically relevant CRPC [9]. As ENZ upregulated PSMA in C4-2 tumors more potently than BIC, it was chosen as the conditioning regimen. Furthermore, it remains to be determined if multiple (vs. single) injections of ^177^Lu-PSMA617 would enhance synergism with a PSMA-inducing drug. As PSMA levels started to decline around days 34–39, testing this hypothesis would have required either (i) to repeat the cycle of ENZ conditioning followed by ^177^Lu-PSMA617 a few weeks after the first cycle or (ii) to continuously administer ENZ until a further injection of ^177^Lu-PSMA617 could safely be given. In the current setting, the prerequisites for a potential positive impact of multiple ^177^Lu-PSMA617 administrations on treatment outcome would include that in scenario (i) subsequent cycles would lead to the same PSMA surge as observed after a single 21-day ENZ regimen and that in scenario (ii) continuous treatment with ENZ would maintain elevated PSMA levels.

Mice in the present study were treated with a single injection of 15 MBq ^177^Lu-PSMA617 (specific activity 84 GBq/μmol) which corresponds to 0.6 GBq/kg body wt in a 25 g mouse. This activity is sub-optimal for treating C4-2 tumors with RLT (our unpublished observations) and should allow the detection of potential synergistic effects of treatment combinations. Clinical protocols currently registered at clinicaltrials.gov use 4–6 cycles ^177^Lu-PSMA with cumulative activities ranging from 14 (4 × 3.7 GBq) to 51 GBq (6 × 8.5 GBq) [27]. In the recently opened prospective phase 3 randomized VISION trial (NCT03511664), patients receive up to 6 cycles with 7.4 ± 10% GBq ^177^Lu-PSMA617 each; assuming an average weight of 80 kg, men thus receive 0.08–0.10 GBq/kg bodyweight ^177^Lu-PSMA617 per cycle. This is 6–7.5 times lower than the activity administered to the mice in our study. However, considering the cumulative activity patients receive over ≤ 6 cycles (maximum 39.96–48.84 GBq in the VISION trial), an 80-kg man would be treated with a total activity of 0.50–0.61 GBq/kg bodyweight. Therefore, the cumulative activity tested in clinical protocols and in our study is similar.

The differences in respective fold increase in PSMA expression measured by flow cytometry vs. ^68^Ga-PSMA11 PET-imaging in this study cannot be explained. However, a limited dynamic range of ^68^Ga-PSMA11 PET signal intensity (acquired with the Genisys 8 PET/CT) has previously been observed [10]. In that study, PSMA expression (as quantified by flow cytometry from FNA tumor biopsies) and ^68^Ga-PSMA PET signal correlated at lower PSMA levels; this correlation was lost at higher PSMA expression levels.

## Conclusion

In summary, we demonstrate that ENZ upregulates PSMA expression in C4-2 tumors that can be monitored non-invasively in vivo by ^68^Ga-PSMA11 PET imaging. PSMA levels peaked at about 3–4 weeks after the start of ARB therapy. ^177^Lu-PSMA617 effectively decreases C4-2 tumor size. Pre-treatment with ARB followed by ^177^Lu-PSMA617 increases DNA damage significantly. However, no additive effect on tumor growth or survival was demonstrated. Clinical studies are needed to determine whether ARB and RLT can yield synergistic effects in patients with CRPC.

## Additional files

Additional file 1: **Figure S1.** PSMA expression kinetics following ARB. C4-2 tumors were induced in Scid mice. Mice received enzalutamide, bicalutamide, or vehicle for 21 days (*n* = 5/group). PSMA expression was estimated by ^68^Ga-PSMA11 PET/CT on days 1, 8, 15, 19, 22, 25, 28, 32, and 36 post start of treatment. Data are represented as mean ± SD. (PDF 32 kb)

Additional file 2: **Figure S2.** Absolute tumor volumes immediately prior to RLT (day 0) and following RLT are shown as mean ± standard deviation for each treatment group. Apparent drops in mean tumor volumes (e.g., vehicle and ENZ-only groups at day 49, RLT-only group at day 74) result from euthanasia of mice that was mandatory due to worsening mouse condition. Usually, these mice were those with the largest tumors in the respective groups. (PDF 29 kb)

Additional file 3: **Table S1.** Fold-change in tumor volume, ^68^Ga-PSMA11 uptake, and PSMA levels as assessed by flow cytometry in C4-2 tumors. Mean ± SD are given. (DOCX 15 kb)

Additional file 4: **Table S2.** Fold-change in phospho-γH2A.X levels following PSMA-RLT. Mean ± SD are given. After 48 h, phospho-γH2A.X levels are significantly higher in the ENZ+RLT groups than in the ENZ-only (*p* = 0.017), RLT-only (*p* = 0.051), and vehicle (*p* < 0.0001) treated groups, respectively. (DOCX 14 kb)

Additional file 5: **Table S3.** Fold-change in C4-2 tumor volume following PSMA-RLT. Mean ± SD are given. (DOCX 14 kb)

## Abbreviations

ARB: Androgen receptor blockade
BIC: Bicalutamide
ENZ: Enzalutamide
FNA: Fine needle aspiration
mCR: Metastatic castration resistant
PC: Prostate cancer
PSMA: Prostate-specific membrane antigen
RLT: Radioligand therapy
RPMI: Rosewell Park Memorial Institute
SD: Standard deviation
