# Supplementary figures and images for: Preclinical evaluation of PSMA expression in response to androgen receptor blockade for theranostics in prostate cancer
Source: EJNMMI Res. 2018 Oct 29;8:96. doi: 10.1186/s13550-018-0451-z (PMC6206308; doi:10.1186/s13550-018-0451-z)

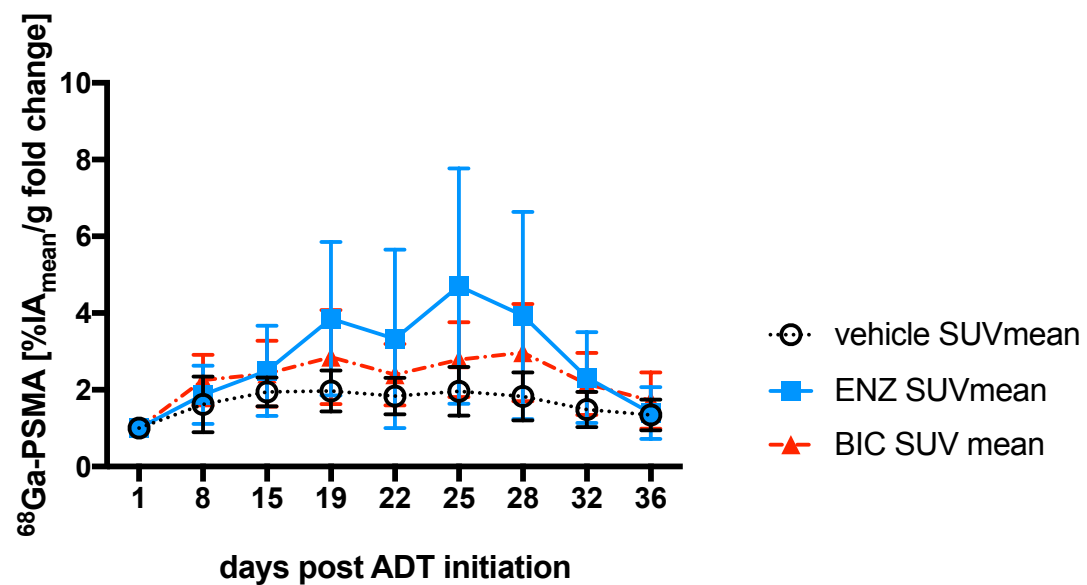

Supplement: Supplementary file 1 — Figure S1. PSMA expression kinetics following ARB. C4-2 tumors were induced in Scid mice. Mice received enzalutamide, bicalutamide, or vehicle for 21 days (n = 5/group). PSMA expression was estimated by 68Ga-PSMA11 PET/CT on days 1, 8, 15, 19, 22, 25, 28, 32, and 36 post start of treatment. Data are represented as mean ± SD. (PDF 32 kb) [file 13550_2018_451_MOESM1_ESM.pdf]

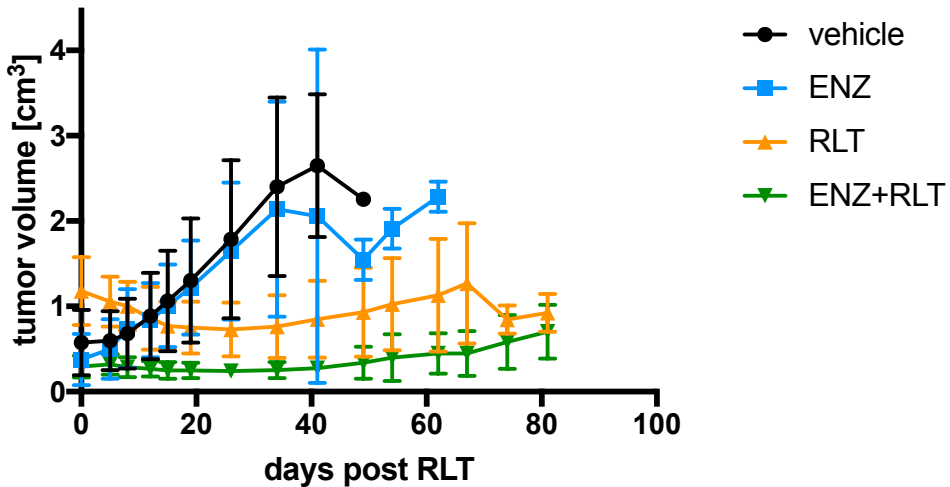

Supplement: Supplementary file 2 — Figure S2. Absolute tumor volumes immediately prior to RLT (day 0) and following RLT are shown as mean ± standard deviation for each treatment group. Apparent drops in mean tumor volumes (e.g., vehicle and ENZ-only groups at day 49, RLT-only group at day 74) result from euthanasia of mice that was mandatory due to worsening mouse condition. Usually, these mice were those with the largest tumors in the respective groups. (PDF 29 kb) [file 13550_2018_451_MOESM2_ESM.pdf]
