# Supplementary material for: Preclinical evaluation of PSMA expression in response to androgen receptor blockade for theranostics in prostate cancer
Source: EJNMMI Res. 2018 Oct 29;8:96. doi: 10.1186/s13550-018-0451-z (PMC6206308; doi:10.1186/s13550-018-0451-z)
Supplement: Supplementary file 3 — Table S1. Fold-change in tumor volume, 68Ga-PSMA11 uptake, and PSMA levels as assessed by flow cytometry in C4-2 tumors. Mean ± SD are given. (DOCX 15 kb) [file 13550_2018_451_MOESM3_ESM.docx]

**Table S1. Fold-change in tumor volume, ^68^Ga-PSMA11 uptake and PSMA levels as assessed by flow cytometry in C4-2 tumors.** Mean±SD are given.

|  | **Time point [day post tumor induction]** | **Tumor volume [mm^3^, fold-change]** | **^68^Ga-PSMA11 PET [%IA_mean_/g, fold-change]** | **^68^Ga-PSMA11 PET [%IA_max_/g, fold-change]** | **PSMA flow cytometry [MFI, fold-change]** |
| --- | --- | --- | --- | --- | --- |
| **C4-2 Control** | **0** | 1.0±0.0 | 1.0±0.0 | 1.0±0.0 | 1.0±0.0 |
|  | **23** | 4.1±2.9 | 3.2±0.9 | 3.1±0.5 | 9.2±1.6 |
|  | **29** | 5.8±4.2 | 2.8±0.4 | 3.0±0.5 | 0.9±0.3 |
|  | **34** | 7.4±6.4 | 3.3±0.6 | 3.7±0.6 | 4.1±3.1 |
|  | **38** | 9.4±9.7 | 2.9±0.9 | 3.1±0.6 | 0.5±0.1 |
|  |  |  |  |  |  |
| **C4-2 ENZ** | **0** | 1.0±0.0 | 1.0±0.0 | 1.0±0.0 | 1.0±0.0 |
|  | **23** | 1.6±0.5 | 7.4±7.2 | 7.0±7.3 | 9.1±5.4 |
|  | **29** | 2.2±1.9 | 4.9±4.0 | 4.3±3.6 | 25.0±21.2 |
|  | **34** | 3.2±2.4 | 4.7±3.6 | 5.0±4.3 | 23.7±26.3 |
|  | **38** | 3.5±2.6 | 4.6±4.4 | 5.4±5.3 | 2.2±2.9 |
|  |  |  |  |  |  |
| **C4-2 BIC** | **0** | 1.0±0.0 | 1.0±0.0 | 1.0±0.0 | 1.0±0.0 |
|  | **23** | 1.1±0.6 | 4.2±2.8 | 3.8±1.4 | 11.8±20.5 |
|  | **29** | 1.4±0.7 | 3.2±1.1 | 3.1±1.2 | 21.8±30.1 |
|  | **34** | 2.2±0.8 | 3.0±0.8 | 3.3±0.9 | 11.5±7.7 |
|  | **38** | 2.9±1.1 | 2.7±1.4 | 3.1±1.6 | 5.1±5.5 |
