# Supplementary material for: Preclinical evaluation of PSMA expression in response to androgen receptor blockade for theranostics in prostate cancer
Source: EJNMMI Res. 2018 Oct 29;8:96. doi: 10.1186/s13550-018-0451-z (PMC6206308; doi:10.1186/s13550-018-0451-z)
Supplement: Supplementary file 4 — Table S2. Fold-change in phospho-γH2A.X levels following PSMA-RLT. Mean ± SD are given. After 48 h, phospho-γH2A.X levels are significantly higher in the ENZ+RLT groups than in the ENZ-only (p = 0.017), RLT-only (p = 0.051), and vehicle (p < 0.0001) treated groups, respectively. (DOCX 14 kb) [file 13550_2018_451_MOESM4_ESM.docx]

**Table S2. Fold-change in phospho-γH2A.X levels following PSMA-RLT.** Mean±SD are given. After 48h, phospho-γH2A.X levels are significantly higher in the ENZ+RLT groups than in the ENZ-only (p=0.017), RLT-only (p=0.051) and vehicle (p<0.0001) treated groups, respectively.

| **Time point [h post RLT]** | **Phospho--γH2A.X** **[% positive cells, fold-change]** | | | |
| --- | --- | --- | --- | --- |
|  | **Control** | **ENZ** | **RLT** | **ENZ+RLT** |
| **0** | 1.0±0.0 | 1.0±0.0 | 1.0±0.0 | 1.0±0.0 |
| **4** | 1.0±0.7 | 1.2±0.5 | 1.2±0.9 | 2.0±1.2 |
| **48** | 1.1±0.4 | 1.8±0.9 | 1.8±1.1 | 3.4±2.2 |
| **96** | 0.9±0.7 | 0.9±0.5 | 1.6±0.8 | 1.5±1.0 |
