# Supplementary material for: Preclinical evaluation of PSMA expression in response to androgen receptor blockade for theranostics in prostate cancer
Source: EJNMMI Res. 2018 Oct 29;8:96. doi: 10.1186/s13550-018-0451-z (PMC6206308; doi:10.1186/s13550-018-0451-z)
Supplement: Supplementary file 5 — Table S3. Fold-change in C4-2 tumor volume following PSMA-RLT. Mean ± SD are given. (DOCX 14 kb) [file 13550_2018_451_MOESM5_ESM.docx]

**Table S3. Fold-change in C4-2 tumor volume following PSMA-RLT.** Mean±SD are given.

| **Time point [day post RLT]** | **Tumor volume [mm^3^, fold-change]** | | | |
| --- | --- | --- | --- | --- |
|  | **Control** | **ENZ** | **RLT** | **ENZ+RLT** |
| **0** | 1.0±0.0 | 1.0±0.0 | 1.0±0.0 | 1.0±0.0 |
| **5** | 1.2±0.3 | 1.6±1.2 | 0.9±0.2 | 1.1±0.2 |
| **8** | 1.3±0.3 | 5.7±11.2 | 0.9±0.1 | 1.7±0.7 |
| **12** | 1.8±0.7 | 3.1±1.9 | 0.7±0.2 | 1.1±0.6 |
| **15** | 2.3±1.0 | 3.7±2.4 | 0.7±0.2 | 0.9±0.2 |
| **19** | 2.9±1.4 | 4.8±3.1 | 0.6±0.2 | 0.9±0.3 |
| **26** | 4.5±2.3 | 7.7±5.1 | 0.6±0.2 | 0.9±0.4 |
| **34** | 7.5±5.9 | 12.7±10.8 | 0.7±0.3 | 1.1±0.9 |
| **41** | 12.7±10.8 | 17.5±10.3 | 0.8±0.4 | 1.3±1.3 |
| **49** |  | 27.9±26.9 | 0.8±0.4 | 1.6±1.9 |
| **54** |  | 35.1±34.3 | 0.9±0.5 | 1.8±1.8 |
| **62** |  | 46.6±50.1 | 0.9±0.4 | 2.1±2.3 |
| **67** |  |  | 1.0±0.4 | 2.2±2.6 |
| **74** |  |  | 0.8±0.2 | 2.9±3.4 |
| **81** |  |  | 0.9±0.2 | 3.7±4.3 |
